# When behavior does not predict glycemic control in older adults with type 2 diabetes: evidence from Lao PDR

**DOI:** 10.3389/fmed.2026.1830071

**Published:** 2026-05-14

**Authors:** Chanmaly Keomalavong, Ranee Wongkongdech

**Affiliations:** 1 Faculty of Medicine, Mahasarakham University, Maha Sarakham, Thailand; 2 Setthathirath Hospital, Vientiane, Laos; 3 International and National Collaborative Network and Innovation for Community Health Development Research Unit (INCNI-CHD), Mahasarakham University, Maha Sarakham, Thailand

**Keywords:** glycemic control, health literacy, Lao PDR, older adults, self-care behavior, type 2 diabetes

## Abstract

**Background:**

Type 2 diabetes mellitus (T2DM) is increasing rapidly in low- and middle-income countries, including Lao People’s Democratic Republic (Lao PDR). Although behavioral self-management is widely considered essential in diabetes care, evidence linking psychosocial determinants to glycemic outcomes among older adults remains inconsistent.

**Objective:**

This study examined the associations between diabetes-related knowledge, attitudes, self-care behaviors, and glycemic control among older adults with T2DM receiving tertiary hospital care in Lao PDR.

**Methods:**

A cross-sectional study was conducted among 88 adults aged ≥60 years with diagnosed T2DM attending the outpatient diabetes clinic at Setthathirath Hospital in Vientiane Capital. Structured interviews were used to assess diabetes knowledge, attitudes, and self-care practices. Glycemic control was defined as HbA1c < 7%. Pearson correlation and multivariable regression analyses were performed to examine associations between psychosocial factors and glycemic outcomes.

**Results:**

A total of 19.3% of participants achieved glycemic control (HbA1c < 7%), with a mean HbA1c level of 9.03 ± 2.47%, indicating generally poor glycemic control. Diabetes knowledge levels were low, with 98.9% of participants classified as having low knowledge. Attitudes toward diabetes management were predominantly low (60.2%), while overall self-care behaviors were largely moderate (83.0%). Pearson correlation analysis showed no statistically significant associations between knowledge (*r* = −0.134, *p* = 0.213), attitudes (*r* = 0.108, *p* = 0.318), or self-care behaviors (*r* = 0.046, *p* = 0.671) and HbA1c levels. Multivariable regression analysis likewise identified no significant predictors of glycemic control.

**Conclusion:**

Despite substantial psychosocial vulnerabilities, no statistically significant associations between psychosocial factors and glycemic control were observed in this sample. These findings may indicate a potential mismatch between psychosocial factors and glycemic outcomes; however, this interpretation should be approached with caution, given the study’s methodological limitations. Further research with larger samples and longitudinal designs is needed to better understand these relationships. This study contributes context-specific evidence from Lao PDR to the limited literature on psychosocial determinants of diabetes management in low- and middle-income countries.

## 1 Introduction

Non-communicable diseases (NCDs) have become a major global health challenge, particularly in low- and middle-income countries undergoing rapid demographic and epidemiological transitions. Diabetes mellitus is one of the most significant contributors to this burden, with global prevalence increasing substantially over recent decades, especially in resource-limited settings (1–3). At the same time, global population aging is accelerating, leading to a growing number of older adults living with chronic diseases such as type 2 diabetes mellitus (T2DM) (4, 5).

Across Southeast Asia, including the Greater Mekong Subregion, rapid urbanization and lifestyle changes have contributed to rising diabetes prevalence, while health systems continue to face limitations in their capacity for chronic disease management (6). In the Lao People’s Democratic Republic (Lao PDR), these challenges are compounded by limited healthcare resources and a relatively low physician-to-population ratio, which may affect long-term management of chronic diseases among aging populations (5).

Effective glycemic control remains central to diabetes management, and psychosocial determinants such as health literacy, diabetes knowledge, and self-care behaviors have traditionally been considered key contributors to metabolic outcomes (7, 8). The Knowledge–Attitude–Practice (KAP) framework posits that knowledge influences attitudes and health behaviors, which, in turn, affect disease outcomes. However, evidence linking behavioral determinants directly to glycemic control remains inconsistent, particularly among older adults with long-standing diabetes (9).

Age-related physiological changes—including progressive beta-cell dysfunction and increasing insulin resistance—may reduce the measurable impact of behavioral factors on metabolic indicators such as HbA1c (10, 11). In addition, healthcare system structures may influence diabetes outcomes. Within structured hospital-based care environments, routine monitoring and pharmacologic management may partially stabilize glycemic control even when psychosocial vulnerabilities remain present (12).

These interacting biological, behavioral, and healthcare system factors suggest the possibility of a clinical–behavioral disconnect, in which metabolic indicators such as HbA1c may not fully reflect underlying psychosocial vulnerabilities among older adults with diabetes. This issue may be particularly relevant in resource-constrained healthcare systems where long-term disease management relies heavily on patient self-care between clinic visits.

Despite the growing burden of diabetes in Lao PDR, empirical evidence examining psychosocial determinants of glycemic control among older adults remains limited. Understanding how these behavioral, biological, and health system factors interact is essential for improving diabetes management in aging populations in resource-constrained healthcare systems. Therefore, this study aimed to examine the associations between diabetes knowledge, attitudes, self-care behaviors, and glycemic control among older adults with type 2 diabetes attending the diabetes clinic at Setthathirath Hospital in Vientiane Capital, Lao PDR.

## 2 Methods

### 2.1 Study design

A cross-sectional study was conducted to examine the associations between psychosocial determinants and glycemic control among older adults with type 2 diabetes mellitus (T2DM). Cross-sectional designs are commonly used to explore behavioral and clinical correlates of diabetes outcomes in real-world healthcare settings, particularly in resource-limited contexts where longitudinal monitoring may be limited (13, 14).

### 2.2 Study setting

The study was conducted at the diabetes clinic of Setthathirath Hospital, a tertiary referral hospital located in Vientiane Capital, Lao PDR. The hospital serves both urban and peri-urban populations and is one of the major public healthcare institutions providing specialized care for chronic diseases, including diabetes.

The diabetes clinic operates as an outpatient service providing routine follow-up care for patients with diabetes. During scheduled visits, patients undergo clinical assessment, including measurement of body weight, blood pressure, and blood glucose levels, followed by a physician consultation to evaluate glycemic control and adjust medication regimens. Patients also receive brief counseling on lifestyle modifications and self-care practices, including diet, physical activity, and medication adherence.

However, diabetes management between clinic visits relies largely on patient self-management. Formal systems for continuous behavioral monitoring, medication reminders, or community-based diabetes support programs remain limited. Patients typically attend follow-up visits every 1-2 months and are expected to manage their condition independently between visits. Such healthcare structures are common in many low- and middle-income countries (LMICs), where clinical care is concentrated in hospital settings and long-term diabetes management relies heavily on patient self-care capacity (2, 6).

### 2.3 Participants and sampling

The required sample size was initially calculated as 502 participants using standard sample size estimation procedures. However, several contextual constraints during the data collection period affected recruitment.

In particular, changes in hospital health insurance policies requiring partial out-of-pocket payments during certain periods reduced clinic attendance among some patients, particularly older adults with financial limitations. Additional barriers such as mobility limitations, transportation challenges, and limited awareness of the importance of regular follow-up care also contributed to lower clinic attendance during the study period.

As a result, 91 participants were recruited. After screening for completeness and data quality, 88 participants were included in the final analysis.

Eligible participants were adults aged 60 years or older with a confirmed diagnosis of type 2 diabetes mellitus who had attended the outpatient diabetes clinic for at least 3 months and had recent HbA1c results available in their medical records.

Participants were recruited consecutively from eligible patients attending the outpatient diabetes clinic during the study period. Due to fluctuations in clinic attendance and the absence of a stable sampling frame, random selection was not feasible. Therefore, the sampling approach more closely reflects consecutive sampling of clinic attendees rather than true random sampling.

### 2.4 Data collection

Data were collected through structured face-to-face interviews using a standardized questionnaire. The instrument assessed key domains related to diabetes self-management, including diabetes-related knowledge, attitudes toward diabetes management, self-care behaviors, and reasons for missed medication doses.

Self-care behaviors included diet, physical activity, sleep, alcohol consumption, smoking, and medication adherence. These psychosocial determinants have been widely recognized as important factors influencing diabetes self-management and glycemic outcomes (7, 8).

In addition, glycated hemoglobin (HbA1c) values were extracted from hospital medical records as an objective indicator of glycemic control.

### 2.5 Measures

The original questionnaire was developed in Thai and translated into English for reporting purposes. Both versions are provided in the Supplementary material, along with detailed item descriptions and variable mapping.

Diabetes knowledge was assessed using a 12-item questionnaire, with correct responses scored as 1 and incorrect or “do not know” responses scored as 0, resulting in total scores ranging from 0 to 12.

Attitudes toward diabetes management were measured using a 14-item Likert scale ranging from 1 (strongly disagree) to 5 (strongly agree). Self-care behaviors were assessed using a 37-item questionnaire covering diet, physical activity, rest, alcohol consumption, smoking, and healthcare-seeking behaviors, with each item rated on a 5-point Likert scale.

Domain-specific scores were calculated by summing item responses, with higher scores indicating better knowledge, more positive attitudes, and more appropriate self-care practices. These scores were further categorized into low, moderate, and high levels based on percentage cut-off points (<60%, 60–79%, and ≥80%).

Glycemic control was defined based on HbA1c levels, with values <7% considered controlled, in accordance with standard clinical guidelines.

### 2.6 Outcome definition

Glycemic control was defined based on HbA1c levels using widely accepted clinical thresholds recommended by international diabetes guidelines:

- Controlled glycemia: HbA1c < 7%
- Uncontrolled glycemia: HbA1c ≥ 7%

HbA1c reflects average blood glucose levels over approximately 3 months and is widely used as a standard indicator of diabetes management outcomes (15).

### 2.7 Statistical analysis

Descriptive statistics were used to summarize participant characteristics and psychosocial variables. Continuous variables were presented as mean ± standard deviation (SD), while categorical variables were summarized as frequencies and percentages.

Pearson correlation analysis was conducted to examine associations between diabetes knowledge, attitudes, self-care behaviors, and HbA1c levels.

Multiple linear regression analysis was performed to assess the combined effects of psychosocial factors and selected demographic variables on HbA1c levels. Statistical significance was defined as *p* < 0.05.

### 2.8 Ethics approval

This study was reviewed and approved by the Ethics Committee of Mahasarakham University (Approval No. 140-045/2568). The research was conducted in accordance with the ethical principles outlined in the Declaration of Helsinki.

Before participation, all eligible participants received a clear explanation of the study objectives, procedures, potential benefits, and possible risks. Written informed consent was obtained from all participants prior to data collection. For older adults who needed assistance understanding the questions, explanations were provided in simple language, and family members were allowed to assist with communication when necessary.

Participation in the study was entirely voluntary. Participants were informed that they could decline participation or withdraw from the study at any time without affecting the quality of their medical care.

All collected data were treated as strictly confidential. Personal identifiers were removed and replaced with coded identification numbers, and results were reported only in aggregated form to ensure that individual participants could not be identified.

## 3 Results

### 3.1 Participant characteristics

A total of 91 older adults with type 2 diabetes were recruited, of whom 88 had complete data for analysis. The mean age of participants was 68.0 ± 6.61 years (range 60–86 years), and slightly more than half were female (53.4%). Most participants had completed primary or secondary education (85.2%), although functional literacy remained limited.

Most participants were married (90.9%) and remained economically active, primarily in small-scale trading and agriculture. Monthly income levels were generally low.

Clinically, more than half of the participants had comorbid hypertension (56.5%), and the mean duration of diabetes was 8.25 ± 5.35 years. Most patients were treated with oral hypoglycemic agents (75.0%), while 25.0% used insulin therapy (Table 1).

**Table 1:** Sociodemographic and clinical characteristics of participants (*n* = 88).

Variable	*n* (%)
Sex (*n* = 87)	
Male	40 (45.5)
Female	47 (53.4)
Age group (*n* = 92)	
60–69 years	59 (64.1)
≥70 years	33 (35.9)
Mean age ± SD	68.0 ± 6.61
Education level (*n* = 88)	
No formal education	4 (4.5)
Primary school	36 (40.9)
Secondary school	39 (44.3)
Diploma or higher	9 (10.2)
Comorbidity (multiple responses allowed)	
Hypertension	52 (56.5)
Other chronic diseases	12 (13.1)
Duration of diabetes (*n* = 88)	
<5 years	36 (40.9)
≥5 years	52 (59.1)
Mean ± SD (years)	8.25 ± 5.35
Treatment type (*n* = 88)	
Oral medication	66 (75.0)
Insulin therapy	22 (25.0)

Denominators vary due to missing data for specific variables; percentages are calculated based on available data for each variable.

### 3.2 Knowledge, attitudes, and self-care behaviors

The overall level of diabetes knowledge among participants was very low, with a mean score of 2.60 ± 2.41 out of a maximum of 12 points. Nearly all participants (98.9%) were classified as having low knowledge levels. Attitudes toward diabetes management were also generally low, with a mean score of 40.41 ± 7.55, and approximately 60.2% of participants demonstrated low attitudes.

In contrast, overall self-care behaviors were moderate, with a mean score of 123.32 ± 12.99. Most participants (83.0%) were categorized as having moderate levels of self-care practices. When examining specific domains of self-care, dietary behaviors were generally poor, whereas physical activity, rest, alcohol consumption, smoking behavior, and healthcare-seeking behaviors were predominantly moderate.

Overall, the findings indicate a marked imbalance between participants’ knowledge and behavior. While knowledge and attitudes about diabetes were generally low, self-care behaviors remained moderate (Tables 2, 3).

**Table 2:** Psychosocial characteristics related to diabetes self-management (*n* = 88).

Variable	Mean ± SD	Low *n* (%)	Moderate *n* (%)	High *n* (%)	Overall level
Knowledge (K)	2.60 ± 2.41	87 (98.9)	1 (1.1)	0 (0.0)	Low
Attitudes (A)	40.41 ± 7.55	53 (60.2)	33 (37.5)	2 (2.3)	Low
Self-care behaviors (P)	123.32 ± 12.99	14 (15.9)	73 (83.0)	1 (1.1)	Moderate
Dietary behaviors	35.78 ± 6.16	48 (54.5)	36 (40.9)	4 (4.5)	Low
Physical activity	10.94 ± 3.35	18 (20.5)	28 (31.8)	42 (47.7)	Moderate
Rest and sleep	20.50 ± 3.91	15 (17.0)	57 (64.8)	16 (18.2)	Moderate
Alcohol consumption	3.88 ± 1.33	17 (19.3)	16 (18.2)	55 (62.5)	Moderate
Smoking behavior	3.91 ± 1.40	20 (22.7)	13 (14.8)	55 (62.5)	Moderate
Health-care seeking behavior	48.31 ± 7.06	11 (12.5)	67 (76.1)	10 (11.4)	Moderate

Missing values may occur due to incomplete responses; percentages are calculated based on available data.

**Table 3:** Summary scores of knowledges, attitudes, and self-care behaviors (*n* = 88).

Variable	Mean ± SD
Diabetes knowledge score	2.60 ± 2.41
Attitude toward diabetes management	40.41 ± 7.55
Self-care behavior score	123.32 ± 12.99

Knowledge scores were based on 12 items (maximum score = 12), attitude scores on 14 items (maximum score = 70), and self-care behavior scores on 37 items (maximum score = 185). Levels were categorized as low (<60%), moderate (60–79%), and high (≥80%) of the total possible score. Alcohol consumption and smoking behaviors were measured using single-item indicators.

Knowledge scores were based on 12 items (maximum score = 12), attitude scores on 14 items (maximum score = 70), and self-care behavior scores on 37 items (maximum score = 185). Levels were categorized by percentage of the total possible score as follows: low (<60%), moderate (60–79%), and high (≥80%).

### 3.3 Glycemic control (HbA1c)

The mean HbA1c level among participants was 9.03 ± 2.47%, indicating generally poor glycemic control. The median HbA1c value was 8.50%, with a range from 5.20 to 18.04%.

When categorized according to standard clinical thresholds, only 19.3% of participants achieved adequate glycemic control (HbA1c < 7%). Approximately one-third of participants (34.1%) had HbA1c levels between 7.0 and 8.9%, while nearly half (46.6%) had HbA1c levels ≥9%, indicating markedly uncontrolled diabetes (Table 4).

**Table 4:** Distribution of HbA1c levels among participants (*n* = 88).

Variable	Value
Mean HbA1c ± SD (%)	9.03 ± 2.47
Median	8.50
Range	5.20–18.04

HbA1c category	*n* (%)
<7% (controlled)	17 (19.3)
7.0–8.9%	30 (34.1)
≥9%	41 (46.6)
Total	88 (100)

HbA1c data were missing for three participants. Percentages are calculated based on available data.

### 3.4 Associations between sociodemographic factors and glycemic control

Chi-square analysis was conducted to examine associations between selected sociodemographic and clinical variables and glycemic control (HbA1c < 7% vs. ≥ 7%). No statistically significant associations were observed between glycemic control and sex, age group, diabetes duration, comorbidities, or medication adherence (all *p* > 0.05).

### 3.5 Associations between psychosocial factors and HbA1c

Pearson correlation analysis showed no statistically significant relationships between diabetes knowledge, attitudes, or overall self-care behaviors and HbA1c levels (Table 5). The correlation between knowledge and HbA1c was weak and negative (*r* = −0.134, *p* = 0.213), whereas attitudes (*r* = 0.108, *p* = 0.318) and self-care behaviors (*r* = 0.046, *p* = 0.671) showed very weak, non-significant associations with glycemic outcomes.

**Table 5:** Associations between psychosocial factors and glycemic control (HbA1c).

Variable	*r*	*p*-value
Diabetes knowledge	−0.134	0.213
Attitude toward diabetes management	0.108	0.318
Self-care behavior	0.046	0.671

Missing values may occur due to incomplete responses; percentages are calculated based on available data.

### 3.6 Multivariable regression analysis

Multiple linear regression was performed to examine the combined effects of diabetes knowledge, attitudes, self-care behaviors, age, and diabetes duration on HbA1c levels. The overall regression model was not statistically significant (*F* = 1.097, *p* = 0.370), explaining only a small proportion of the variance in HbA1c (*R*^2^ = 0.073; adjusted *R*^2^ = 0.006). None of the independent variables was significantly associated with HbA1c levels.

Additional analysis examining medication forgetting also showed no significant relationship with HbA1c (*B* = 0.244, *p* = 0.224; *R*^2^ = 0.017). Interaction analysis between diabetes knowledge and disease duration likewise did not demonstrate a significant moderating effect on HbA1c (*B* = 0.035, *p* = 0.255).

Overall, the results indicated substantial gaps in diabetes-related knowledge among older adults with type 2 diabetes in this tertiary care setting. Despite low levels of knowledge and predominantly moderate self-care behaviors, glycemic control remained poor, with a mean HbA1c of 9.03%. However, statistical analyses consistently showed no statistically significants between knowledge, attitudes, self-care behaviors, and glycemic outcomes. Both correlation and regression analyses demonstrated weak, non-significant relationships between psychosocial variables and HbA1c levels, suggesting that behavioral factors alone may not fully explain metabolic outcomes in this population.

## 4 Discussion

The present study addresses an important clinical question regarding the relationship between psychosocial determinants and glycemic outcomes among older adults with type 2 diabetes. The findings suggest a potential mismatch between psychosocial factors and glycemic control in this tertiary care setting. Despite substantial psychosocial vulnerabilities—including limited diabetes knowledge and motivational barriers toward medication adherence—these factors were not significantly associated with HbA1c levels in this sample (7, 16). The absence of statistically significant associations may partly be attributable to limited statistical power due to the relatively small analytic sample size. In addition, the limited variability and skewed distributions observed in several psychosocial variables may have reduced the analyses’ sensitivity to detect statistically significant associations, even if they exist.

One possible explanation for this finding is the broader system gap between clinical care and daily self-management in diabetes. In the study setting, clinical care is primarily delivered through scheduled outpatient visits, during which patients receive clinical assessment, medication adjustment, and brief counseling. However, during the intervals between clinic visits, diabetes management relies largely on patients’ ability to manage their condition independently in daily life. In the absence of structured follow-up mechanisms—such as behavioral monitoring, medication reminders, or community-based support—patients may struggle to translate medical advice into sustained self-care behaviors. This gap between clinic-based care and everyday self-management may partly explain the observed weak associations between psychosocial determinants and glycemic outcomes in this population.

The present findings contribute to the growing body of evidence suggesting that metabolic indicators such as HbA1c may not fully capture psychosocial vulnerability among older adults with diabetes, particularly in structured clinical care environments. Previous research has frequently emphasized the importance of diabetes knowledge, health literacy, and self-care behaviors in achieving optimal glycemic outcomes (7, 8). Diabetes self-management education programs have been shown to improve glycemic control in many settings, particularly when patients demonstrate adequate understanding of disease management (9, 17, 18). However, the strength of this association varies across populations, and several studies have reported inconsistent relationships between psychosocial determinants and HbA1c, particularly among older adults and individuals with long-standing diabetes.

The conceptual model illustrates how psychosocial determinants—such as diabetes knowledge, attitudes, and self-care behaviors—are theoretically expected to influence glycemic control through patient self-management. However, the present findings showed weak and non-significant associations between these psychosocial variables and HbA1c levels. These findings may suggest that glycemic outcomes in this setting are not strongly associated with the measured psychosocial factors and may be influenced by other unmeasured factors, rather than psychosocial variables alone, highlighting a potential clinical–behavioral disconnect among older adults with type 2 diabetes. The structured nature of tertiary hospital care may also play an important role. Patients receiving care in hospital-based diabetes clinics often benefit from regular clinical monitoring, medication titration, and standardized follow-up. Such structured care systems may partially stabilize glycemic outcomes through pharmacologic intensification and professional oversight, consistent with the Chronic Care Model (12, 19). Under these circumstances, glycemic control may be partially maintained through structured clinical management, even in the presence of limitations in patient knowledge or motivation.

These findings are particularly relevant in low- and middle-income countries (LMICs), where health literacy and diabetes education resources may be limited (3, 6). Studies from Southeast Asia and other resource-constrained settings have reported substantial gaps in knowledge of diabetes and in self-management practices among older adults (13, 14). However, healthcare system factors—including structured clinical follow-up and pharmacological management—may partially mitigate the impact of behavioral limitations on metabolic outcomes.

**Figure 1:**
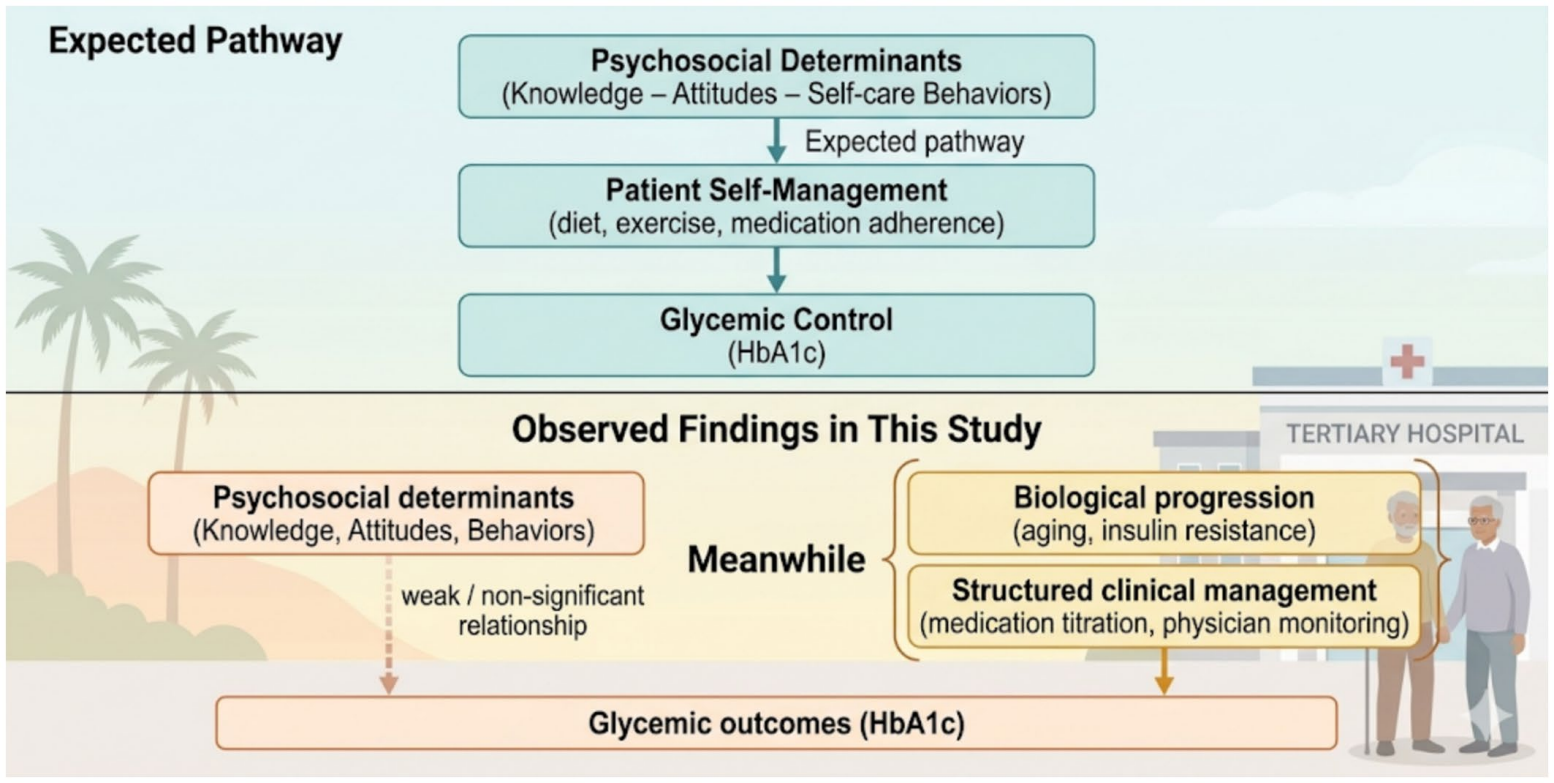
Conceptual model of the clinical–behavioral disconnect in diabetes management among older adults with type 2 diabetes in Lao PDR.

Another explanation relates to the biological progression of diabetes in aging populations. Age-related decline in pancreatic beta-cell function, increasing insulin resistance, and longer disease duration may attenuate the measurable influence of behavioral differences on metabolic indicators such as HbA1c (10, 11). In older adults, physiological factors may contribute substantially to glycemic outcomes, potentially attenuating the observable effects of psychosocial variables. Figure 1 illustrates the conceptual model of the clinical–behavioral disconnect identified in this study.

The upper panel illustrates the expected theoretical pathway in which psychosocial determinants—including diabetes knowledge, attitudes, and self-care behaviors—influence glycemic control through patient self-management. However, the lower panel shows the findings of the present study, in which psychosocial factors showed weak, non-significant associations with HbA1c levels. Instead, glycemic outcomes in this tertiary care setting may be influenced by factors not measured in this study, including potential biological and clinical factors.

### 4.1 Regional significance of Lao data

Evidence on diabetes management among older adults in Lao PDR remains extremely limited, and most available data on psychosocial determinants of glycemic control originate from higher-income countries or larger middle-income countries in Southeast Asia. As a result, understanding how behavioral, clinical, and health system factors interact in smaller and resource-constrained settings remains an important research gap.

The present study contributes valuable empirical evidence from the Lao PDR, where the burden of non-communicable diseases (NCDs) has increased rapidly over the past two decades due to demographic aging, urbanization, and lifestyle changes (2). Similar challenges have been reported across Southeast Asia, where diabetes prevalence has risen substantially while health systems continue to adapt to the growing demand for chronic disease management (6).

By examining psychosocial factors alongside clinical outcomes among older adults receiving tertiary hospital care, this study provides important insights into diabetes management within the Lao healthcare system. The findings suggest that the relationship between psychosocial factors and glycemic outcomes may be more complex and less consistent than commonly assumed, particularly in resource-constrained healthcare settings. These results highlight the need for future research exploring how behavioral interventions, patient education programs, and community-based diabetes support systems can be integrated into existing healthcare structures in Lao PDR and similar low- and middle-income country settings.

### 4.2 Public health implications

The findings of this study have important implications for diabetes management in resource-limited settings such as Lao PDR. Although structured clinical care in tertiary hospitals may contribute to stabilizing metabolic outcomes, substantial psychosocial vulnerabilities among older adults—including limited diabetes knowledge and motivational barriers to medication adherence—remain evident. These findings suggest that glycemic indicators alone may not adequately capture patients’ broader behavioral and educational needs.

From a health system perspective, strengthening diabetes education and behavioral support should become a priority within national non-communicable disease (NCD) control strategies. In the Lao PDR, the growing burden of diabetes and other NCDs has been recognized as a major public health challenge, particularly amid rapid demographic and lifestyle transitions (2, 3). Integrating structured diabetes self-management education into routine care has been shown to improve glycemic outcomes and patient engagement (17, 18), and could therefore be supported through collaboration between the Ministry of Health, the Department of Hygiene and Health Promotion, and hospital-based chronic disease clinics.

Strengthening patient education programs within tertiary and secondary healthcare facilities, alongside improved community-based follow-up through primary healthcare networks, may enhance long-term diabetes self-management capacity among older adults. Evidence from Southeast Asia suggests that limited health literacy, inadequate access to diabetes education, and gaps in patient-centered care remain key barriers to effective diabetes management in low- and middle-income countries (13, 14, 16).

In addition, national NCD initiatives supported by international partners such as the World Health Organization (WHO) and the International Diabetes Federation (IDF) could play an important role in strengthening diabetes literacy and behavioral support systems. Integrating psychosocial assessment, counseling services, and culturally appropriate health education into routine diabetes care may help address the gap between clinical management and patient self-management capacity. Such efforts align with the WHO Global Action Plan for the Prevention and Control of Noncommunicable Diseases and may help improve long-term diabetes outcomes and reduce complications in aging populations in Lao PDR and similar low- and middle-income country settings (6, 20). In addition, the findings contribute to ongoing discussions in the literature regarding the inconsistent relationship between behavioral determinants and glycemic outcomes, particularly in aging populations and structured clinical care settings.

### 4.3 Limitations

This study has several limitations. First, the cross-sectional design limits the ability to establish causal relationships between psychosocial determinants and glycemic outcomes. Second, the relatively modest sample size (*n* = 88), compared to the originally planned sample may have reduced statistical power to detect independent predictors in multivariable analyses and increased the likelihood of type II error. This reduction in sample size was partly due to contextual factors affecting clinic attendance during the study period, as described in the Methods section. Third, behavioral variables were measured using self-reported instruments and may therefore be subject to recall or reporting bias. In addition, the use of consecutive sampling may have introduced selection bias, as the sample may not be fully representative of the broader population of older adults with type 2 diabetes in this setting. In addition, several variables demonstrated limited variability and skewed distributions, which may have further reduced the ability to detect statistically significant associations. These methodological and measurement limitations should be considered when interpreting the findings. Despite these limitations, the study provides rare empirical evidence on psychosocial determinants of diabetes management among older adults in Lao PDR, a context where clinical and behavioral research on diabetes remains scarce.

## 5 Conclusion

Among older adults with type 2 diabetes receiving care at Setthathirath Hospital in Vientiane Capital, substantial deficits in diabetes knowledge and motivational barriers to medication adherence were observed. However, no statistically significant associations were found between psychosocial factors and glycemic control within this sample.

These findings may indicate a potential mismatch between psychosocial factors and glycemic outcomes in this setting; however, this interpretation should be approached with caution, given the study’s methodological limitations, including sample size, measurement characteristics, and study design.

Overall, the results highlight the complexity of diabetes management in aging populations and suggest that metabolic indicators alone may not fully capture patients’ behavioral and psychosocial factors. Future research with larger samples and longitudinal designs is needed to better understand these relationships. Strengthening patient education, psychosocial support, and integrated care strategies may help improve long-term diabetes outcomes in Lao PDR and similar low- and middle-income country settings.

This study contributes to the literature by providing context-specific empirical evidence from an understudied setting, suggesting that the relationship between psychosocial determinants and glycemic outcomes may be more complex and less consistent than commonly assumed, particularly among older adults in resource-constrained healthcare systems.

## Data Availability

The raw data supporting the conclusions of this article will be made available by the authors, without undue reservation.
