# Supplementary material for: When behavior does not predict glycemic control in older adults with type 2 diabetes: evidence from Lao PDR
Source: Front Med (Lausanne). 2026 May 14;13:1830071. doi: 10.3389/fmed.2026.1830071 (PMC13215864; doi:10.3389/fmed.2026.1830071)
Supplement: Supplementary file 1 [file Data_Sheet_1.pdf]

Supplementary File 1

Structured Questionnaire and Variable Mapping (KAP Study)

Instructions: Please tick (✓) or fill in the appropriate answer based on your actual situation.

Part 1: General Information

1. Age (years)
2. Sex (Male/Female)
3. Education level
4. Marital status
5. Occupation
6. Monthly income
7. Monthly expenses
8. Fasting blood glucose (recent)
9. Duration of diabetes

Part 2: Knowledge about Diabetes (12 items)

1. Diabetes is caused by consuming sugary foods.
2. Diabetes can be hereditary.
3. Diabetes is related to health behaviors.
4. Diabetes can be completely cured.
5. Symptoms include fatigue, thirst, and frequent urination.
6. Blood glucose should be regularly monitored.
7. Poor control leads to complications.
8. Eating very little helps control diabetes.
9. Eating vegetables helps control diabetes.
10. Fruits can be consumed without restriction.
11. Carbohydrates affect blood glucose the most.
12. Regular exercise helps control diabetes.

Part 3: Attitudes (Likert Scale 1–5)

1. Following dietary advice helps control blood sugar.
2. Reducing carbohydrates improves control.
3. Sweets should be avoided.
4. Medication is necessary.
5. Lifestyle changes are important.
6. I feel confident managing diabetes.
7. I worry about complications.
8. Regular monitoring is beneficial.

Part 4: Self-care Behaviors (Likert Scale 1–5)

1. Follow recommended diet
2. Reduce sugar intake
3. Eat vegetables regularly
4. Exercise regularly
5. Take medication as prescribed
6. Attend follow-up visits

7. Monitor blood glucose
8. Avoid smoking
9. Limit alcohol
10. Do not adjust medication without advice

Scoring System

Knowledge: Correct=1, Incorrect/Don't know=0
Attitudes: Likert scale (1–5), higher scores = positive attitudes
Behaviors: Likert scale (1–5), higher scores = better self-care

Supplementary Table S1: Mapping of Questionnaire Items to Variables

Domain	Variable	Items	Scale	Scoring	Interpretation
Knowledge	General knowledge	1–12	Binary	1/0	Higher=better
Attitudes	Perception	1–8	Likert 1–5	Sum	Higher=positive
Behavior	Self-care	1–10	Likert 1–5	Sum	Higher=better
Outcome	Glycemic control	FBS	Clinical	Categorized	Controlled vs uncontrolled
