# Supplementary material for: When behavior does not predict glycemic control in older adults with type 2 diabetes: evidence from Lao PDR
Source: Front Med (Lausanne). 2026 May 14;13:1830071. doi: 10.3389/fmed.2026.1830071 (PMC13215864; doi:10.3389/fmed.2026.1830071)
Supplement: Supplementary file 2 [file Data_Sheet_2.pdf]

แบบสอบถาม

การศึกษาหา ความรู้ ทัศนคติ และ พฤติกรรม ด้านสุขภาพ ในผู้สูงอายุที่ป่วยเป็นโรคเบาหวานชนิดที่ 2 ที่มารับบริการที่แผนกผู้ป่วยนอก คลินิกเบาหวาน โรงพยาบาลเชษฐาธิราช สาธารณรัฐประชาธิปไตย ประชาชนลาว

รหัสแบบสอบถาม: ☐☐☐☐☐

วันที เดือน ปี:................../..................../.....................

ผู้สำพาด...

ส่วนที่ 1 ข้อมูลพื้นฐานทั่วไปของผู้ตอบแบบสอบถาม

คำชี้แจง โปรดทำเครื่องหมาย √ ลงใน ☐ หรือเติมข้อความลงในช่องว่างตามความเป็นจริง

1. อายุ.................................. ปี
2. เพศ
   - ☐ ชาย
   - ☐ หญิง
3. ระดับการศึกษา
   - ☐ ไม่ได้เข้าโรงเรียน
   - ☐ ประถมศึกษา
   - ☐ มัธยมศึกษา
   - ☐ อนุปริญญา
   - ☐ ปริญญาตรี หรือ สูงกว่า
4. สถานภาพสมรส
   - ☐ โสด
   - ☐ สมรส
   - ☐ หย่าร้าง
   - ☐ หม้าย
5. ประกอบอาชีพหลัก
   - ☐ ข้าราชการ
   - ☐ รับจ้าง

☐ ค้าขาย

☐ ประกอบอาชีพอิสระ

☐ เกษตรกร

☐ อื่นๆ...........................

6. รายได้ต่อเดือน

☐ ≤1,000 บาท

☐ 1,001 – 3,000 บาท

☐ 3,001 – 6,000 บาท

☐ 6,001 – 10,000 บาท

☐ >10,000 บาท

7. ค่าใช้จ่ายเฉลี่ยต่อเดือน

☐ ≤1,000 บาท

☐ 1,001 – 3,000 บาท

☐ 3,001 – 6,000 บาท

☐ 6,001 – 10,000 บาท

☐ >10,000 บาท

8. ระดับน้ำตาลในเลือดก่อนอาหารเช้า หลังจากงดน้ำงดอาหารเป็นเวลา 8 ชั่วโมง (ภายในเดือนที่มีการสัมภาษณ์)

☐ ≤70 mg/dl

☐ 71 – 110 mg/dl

☐ 111 – 150 mg/dl

☐ 151 – 180 mg/dl

☐ > 180 mg/dl

9. ระยะเวลาในการป่วยเป็นโรคเบาหวาน

☐ หน้อยกว่า 1 ปี

☐ 1 ปีขึ้นไป หา 3 ปี

☐ 3 ปีขึ้นไป หา 5 ปี

☐ 5 ปีขึ้นไป

ส่วนที่ 2 แบบทดสอบความรู้ทั่วไปเกี่ยวกับโรคเบาหวาน

คำชี้แจง โปรดทำเครื่องหมาย √ ใน ☐ หรือ ในช่องที่ตรงตามความคิดเห็นของท่านมากที่สุด

10. ท่านรู้หรือเคยได้ยินเกี่ยวกับโรคเบาหวานหรือไม่? (ถ้าไม่ ข้ามไปที่หัวข้อที่ 12)

    ☐ รู้

    ☐ ไม่รู้

    ☐ ไม่แน่ใจ

11. ถ้ารู้ ท่านรู้หรือเคยได้ยินเกี่ยวกับโรคเบาหวานมาจากที่ไหน? (ตอบได้มากกว่าหนึ่งข้อ)

    ☐ แพทย์ หมอ

    ☐ พยาบาล

    ☐ เจ้าหน้าที่สาธารณสุข

    ☐ เพื่อน

    ☐ สืออออนไลน์

    ☐ อื่นๆ...........................

12. ผู้เป็นเบาหวานควรมีระดับน้ำตาลในเลือดก่อนรับประทานอาหารอยู่ในช่วงระดับเท่าไร?

    ☐ ≤70 mg/dl

    ☐ 71 – 110 mg/dl

    ☐ 111 – 150 mg/dl

    ☐ 151 – 180 mg/dl

    ☐ > 180 mg/dl

    ☐ ไม่ทราบข้อมูล

13. ผู้เป็นเบาหวานสามาถตรวจวัดระดับน้ำตาลในเลือดด้วยตนเองที่บ้านเพื่อติดตามระดับน้ำตาลได้หรือไม่?

    ☐ ได้

    ☐ ไม่ได้

    ☐ ไม่ทราบข้อมูล

ความรู้ทั่วไปเกี่ยวกับโรคเบาหวาน	คำตอบ		
	ใช่	ไม่แน่ใจ	ไม่ใช่
14. สาเหตุของโรคเบาหวาน เกิดจากการกินอาหารหวานหรืออาหารที่มีน้ำตาลมาก			
15. สาเหตุของโรคเบาหวาน เกิดจากกรรมพันธุ์			
16. สาเหตุของโรคเบาหวาน เกิดจากพฤติกรรมการดูแลสุขภาพของตนเอง			
17. โรคเบาหวานสามารถรักษาให้หายขาดได้			
18. อาการของโรคเบาหวาน ได้แก่ อ่อนเพลีย หิวบ่อย กระหายน้ำบ่อย ปัสสาวะบ่อย และ น้ำหนักตัวลดลง			
19. ผู้ป่วยเบาหวานต้องตรวจระดับน้ำตาลในเลือดเป็นประจำ			
20. หากควบคุมระดับน้ำตาลในเลือดได้ไม่ดี จะทำให้เกิดภาวะแทรกซ้อนเรื้อรังต่างๆ ได้แก่ ไตวายเรื้อรัง กล้ามเนื้อหัวใจขาดเลือด อัมพาต ชาปลายเท้า และเบาหวานขึ้นตา			
21. ผู้เป็นเบาหวานควรอดอาหารหรือกินข้าวเพียง 2 – 3 คำต่อมื้อ จะช่วยให้ควบคุม เบาหวานได้ดีขึ้น			
22. เป็นเบาหวานควรกินผักทุกวัน อย่างน้อยมื้อละ 1 ทัพพี โดยเน้นผักใบให้มาก เช่น ผักบุ้ง เพื่อช่วยให้ควบคุม เบาหวานได้ดีขึ้น			
23. ผู้เป็นเบาหวานสามารถกินผลไม้รสไม่หวานจัด เช่น ส้ม ฝรั่ง ชมพู่ แอปเปิ้ล เป็นต้น ปริมาณเท่าใดก็ได้โดยไม่ต้องจำกัดปริมาณ			
24. อาหารที่มีผลต่อระดับน้ำตาลในเลือดมากที่สุดคือ อาหารที่มีคาร์โบไฮเดรต			
25. หากลืมกินยามื้อใดมื้อหนึ่ง สามารถกินเพิ่มเป็น 2 เท่าในมื้อถัดไปได้			
26. การสูบบุหรี่หรือดื่มเครื่องดื่มแอลกอฮอล์ไม่มีผลในการควบคุมโรคเบาหวานแต่อย่างใด			
27. เป็นเบาหวานควรออกกำลังกายด้วยการยืดเหยียดกล้ามเนื้อ แกว่งแขน เดิน อย่างน้อย ครั้งละ 30 นาที สัปดาห์ละ 3 – 5 วัน หรืออย่างน้อย 150 นาที/สัปดาห์			
28. ผู้ป่วยเบาหวานต้องดูแลร่างกายโดยเฉพาะอย่างยิ่ง เท้าและผิวหนังไม่ให้เกิดบาดแผลเป็นอันขาด			

ส่วนที่ 3 แบบประเมินทัศนคติเกี่ยวกับโรคเบาหวาน

คำชี้แจง โปรดทำเครื่องหมาย √ ในช่องที่ตรงตามความคิดเห็นของท่านมากที่สุด ตามลำดับดังนี้:

1 ไม่เห็นด้วยอย่างยิง

2 ไม่เห็นด้วย

3 ไม่แน่ใจ

4 เห็นด้วย

5 เห็นด้วยอย่างยิง

ทัศนคติเกี่ยวกับโรคเบาหวาน	1	2	3	4	5
29. ฉันคิดว่า ถ้ากินอาหารตามคำแนะนำของหมอ อย่างเคร่งครัด สามาถควบคุมน้ำตาลในเลือด					
30. ฉันคิดว่า การหลีกเลี่ยงกินข้าว/แป้ง หรือกินให้น้อยลง แล้ว กินผักให้มาก สามาถทำให้ควบคุมน้ำตาลในเลือดได้ดีขึ้น					
31. ฉันคิดว่า ผลไม้ที่มีรสเปรี้ยว เช่น สับปะรด มะเฟือง มะม่วง และลิ้นจี่ เป็นต้น สามารถรับประทานได้มากเท่าใดก็ได้					
32. ฉันคิดว่า ผู้เป็นเบาหวานไม่ควรกินขนมหวาน เช่น บัวลอย กล้วยบวดชี เป็นต้น หรือ กินนานๆครั้ง					
33. ฉันคิดว่า ถ้ากินยารักษาเบาหวานก็ไม่จำเป็นต้องควบคุม อาหารหรือออกกำลังกายอีก					
34. ฉันคิดว่า ถ้าควบคุมน้ำตาลได้ดีแล้วก็ไม่จำเป็นต้องกินยา สม่ำเสมอ กินบ้าง หยุดบ้างก็ได้ไม่เป็นอันตรายอะไร					
35. ฉันคิดว่า การกินยารักษาเบาหวานเป็นเวลานานมีส่วนทำให้ เป็นโรคตับหรือ โรคไตได้					
36. ฉันคิดว่า การเจาะเลือดปลายนิ้วด้วยตนเองเป็นประจำส่งผล ให้ควบคุมน้ำตาล ในเลือดได้ดีขึ้น					
37. ฉันคิดว่า หากควบคุมน้ำตาลในเลือดได้ตามเป้าหมาย จะช่วย ชะลอการเกิด ภาวะแทรกซ้อนเรื้อรัง เช่น เบาหวานขึ้นตา ไต วายเรื้อรัง เป็นต้น					
38. ฉันคิดว่า การออกกำลังกายอย่างอย่างสม่ำเสมอทำให้การ ควบคุมเบาหวานได้ดีขึ้น					

ส่วนที่ 4 แบบประเมินพฤติกรรมการควบคุมโรคเบาหวาน

คำชี้แจง โปรดทำเครื่องหมาย √ ในช่องที่ตรงตามความคิดเห็นของท่านมากที่สุด ตามลำดับดังนี้:

1 ไม่เคยปฏิบัติเลย

2 ปฏิบัติน้อย

3 ปฏิบัติปานกลาง

4 ปฏิบัติมาก

5 ปฏิบัติเป็นประจำ

พฤติกรรมการควบคุมโรคเบาหวาน	1	2	3	4	5
39. ท่านรับประทานอาหารประเภทข้าวเหนียว					
40. ท่านรับประทานอาหารประเภทข้าวสวย					
41. ท่านรับประทานอาหารไม่เป็นเวลา เมื่อหิวจึงรับประทาน					
42. ท่านรับประทานอาหารประเภทอาหารที่มีไขมันสูง หรืออาหารประเภททอด					
43. ท่านรับประทานอาหารประเภทผักต่างๆ					
44. ท่านรับประทานอาหาร และ ผลไม้ที่มีรสหวาน					
45. ท่านดื่มน้ำอัดลม น้ำหวาน น้ำผลไม้					
46. ท่านดื่มสุรา เบียร์หรือเครื่องดื่มที่มีแอลกอฮอล์					
47. ท่านสูบบุหรี่					
48. ท่านออกกำลังกายอย่างต่อเนื่องอย่างนอ้ยครั้งละ 30 นาที และ ไม่หักโหมจนเกินไป					
49. ท่านออกกำลังกายเช่น เดิน/วิ่ง เต้นแอโรบิค ปั่นจักรยาน					
50. ท่านใส่รองเท้าที่เหมาะสมในการออกกำลังกายและสวมถุงเท้าด้วยทุกครั้ง					
51. ท่านได้เข้าพบหมอตามนัดหมายอย่างเคร่งครัด					
52. ท่านได้ตรวจวัดระดับน้ำตาลของตนเองที่บ้าน					
53. ท่านรับประทานยาเบาหวานตรงตามเวลาและปริมารตามแพทย์สั่ง					
54. ท่านได้อ่านฉลากยาให้เข้าใจ และ ตรวจสอบวันหมดอายุของยาก่อนรับประทานยาทุกครั้ง					

55. ท่านปรับขนาดยาเบาหวานโดยลดหรือเพิ่มยาหรือหยุดยาด้วยตัวเอง					
56. ถ้าท่านลืมรับประทานยาเบาหวานท่านจะรับประทานยาทันที ที่นึกขึ้นได้					
57. เมื่อมีอาการไม่สบายเช่น เป็นไข้หวัด เจ็บคอ ท่านจะงดยาเบาหวานในวันนั้น					
